# A Multiplex Liquid Array Assay Based on Luminex xTAG Technology for the Detection of Five Respiratory Pathogens in SPF Chickens

**DOI:** 10.3390/vetsci13080753

**Published:** 2026-07-29

**Authors:** Qiuyang Sun, Yufang Feng, Fangwei Dai, Qiang Gao, Xueqing Zhang, Shanshan Yao, Rui Fu, Chunnan Liang, Jin Xing

**Affiliations:** 1 Institute of Laboratory Animal Resources (National Rodent Experimental Animal Resource Bank), National Institutes for Drug Control, Beijing 102629, China; qiyang07@outlook.com (Q.S.); fengyufang@nifdc.org.cn (Y.F.); gaoqiang@nifdc.org.cn (Q.G.); zhangxueqing@nifdc.org.cn (X.Z.); yaoshanshan@nifdc.org.cn (S.Y.); furui78@nifdc.org.cn (R.F.); chunnan_liang@nifdc.org.cn (C.L.); 2 Center of Laboratory Animal, Hangzhou Medical College, Hangzhou 310013, China; fangweidai@163.com

**Keywords:** SPF chickens, Luminex xTAG-based liquid array, *Mycoplasma gallisepticum*, *Mycoplasma synoviae*, *Pasteurella multocida*, *Avibacterium paragallinarum*, *Mycobacterium avium*

## Abstract

A multiplex liquid array assay based on Luminex xTAG technology was developed for the first time for the simultaneous detection of five respiratory bacterial pathogens in Specific Pathogen-Free (SPF) chickens—*Mycoplasma gallisepticum* (*MG*), *Mycoplasma synoviae* (*MS*), *Pasteurella multocida* (*Pm*), *Avibacterium paragallinarum* (*Apg*), and *Mycobacterium avium* (*Mav*)—filling the gap that these five pathogens could not be simultaneously detected previously. Specific primers were designed targeting conserved genes of each pathogen, with a TAG sequence attached to the 5′ end of each forward primer and biotin to the 5′ end of each reverse primer. After multiplex PCR amplification, the products were hybridized with MagPlex-TAG microspheres and analyzed using the Luminex system. The method was validated for sensitivity, specificity, and repeatability, and tested with simulated positive samples (a mixture of genomic DNA from the five target pathogens) and SPF chicken samples. The LOD was 10 copies/μL for *Pm* and 100 copies/μL for the other four pathogens. No cross-reactivity was observed with 18 non-target bacterial species. Intra- and Inter-assay coefficients of variation (CV) ranged from 0.8% to 12.2% and 2.3% to 14.4%, respectively, both below 15%. All simulated mixed positive samples yielded results consistent with expectations, and none of the 114 nasopharyngeal swab samples from SPF chickens tested positive for any of the five targets. The developed assay offers high throughput, high sensitivity, high specificity, and good repeatability, providing a robust tool for routine quality control in SPF chickens.

## Simple Summary

Specific pathogen-free (SPF) chickens are essential for the reliability and reproducibility of scientific research data. Among the pathogens that must be excluded, five respiratory bacterial species are of particular importance. Currently, the lack of a unified testing method—with existing assays covering anywhere from two to four targets—makes the process time-consuming and cost-inefficient. In this study, we developed a new method capable of simultaneously detecting all five bacteria within a single reaction system. The assay functions by detecting specific target genes unique to each bacterium. Our results demonstrated that this method exhibits high sensitivity, specificity, and repeatability. It can accurately identify the target bacteria and shows no cross-reactivity with other common bacterial species. When we tested samples from SPF chickens, none of the five bacteria were detected, as expected. This method provides a faster, simpler, and more efficient tool for routine monitoring of SPF chickens, thereby contributing to improved quality control and more reliable research data.

## 1. Introduction

Laboratory animals are indispensable resources in life science research, and their microbiological quality control directly affects the accuracy and reproducibility of scientific data. Specific pathogen-free (SPF) chickens serve as important animal models in various fields of life science research. According to the Chinese national standard GB/T 17999.1-2008 [1] and the Chinese Pharmacopoeia (2025 Edition, Volume IV) [2], SPF chickens must be verified as free from five respiratory bacterial pathogens: *Mycoplasma gallisepticum* (*MG*), *Mycoplasma synoviae* (*MS*), *Pasteurella multocida* (*Pm*), *Avibacterium paragallinarum* (*Apg*), and *Mycobacterium avium* (*Mav*).

These five pathogens pose significant threats to poultry health and are of particular concern in SPF chickens. *MG* causes chronic respiratory disease, reduced egg production, and poor feed conversion [3]; *MS* causes infectious synovitis (joint swelling and lameness) and respiratory disease [4]; *Pm* causes fowl cholera, which can lead to acute septicemia and high mortality [5]; *Apg* causes infectious coryza, characterized by nasal discharge, facial swelling, and decreased egg production [6]; and *Mav* causes avian tuberculosis, a chronic wasting disease leading to emaciation and reduced productivity [7]. Importantly, *MG* and *MS* are vertically transmitted through eggs, making their exclusion critical for SPF chickens [8]. Furthermore, the presence of any of these pathogens can interfere with experimental outcomes by inducing immune responses, altering baseline physiological parameters, or confounding challenge studies, thereby compromising data reproducibility and reliability [9].

Traditionally, the detection of these five pathogens has relied on culture isolation and biochemical identification. However, these pathogens have diverse growth requirements and are generally slow-growing, making conventional methods time-consuming (often taking several weeks) and operationally cumbersome, which cannot meet the timeliness requirements for routine health monitoring of SPF chickens.

With the development of molecular biology techniques, PCR-based detection methods have gradually become important tools for pathogen detection due to their rapidity and high sensitivity, and their application formats have evolved from single-target PCR to multiplex PCR and then to real-time fluorescence quantitative PCR (qPCR). Single-target PCR can achieve species-specific detection but requires separate reactions for each pathogen, resulting in low efficiency; conventional multiplex PCR can simultaneously detect multiple targets in a single reaction but is limited by primer compatibility issues and gel-based endpoint detection; qPCR significantly improves sensitivity and quantification capability, yet its multiplexing capacity is constrained by the number of available fluorescence channels, typically allowing only 4–6 targets per reaction. For SPF chicken respiratory pathogens, researchers have developed various molecular detection methods, including single-plex or multiplex qPCR [10,11,12] and conventional multiplex PCR [13,14]; commercial kits are also available. However, these methods still fall short of meeting the requirement for simultaneous detection of all five respiratory bacterial pathogens (*MG*, *MS*, *Pm*, *Apg*, and *Mav*) in a single reaction, and remain inadequate in terms of detection throughput, cost-effectiveness, and target coverage completeness.

Luminex xTAG liquid array technology, based on fluorescently coded microspheres, offers a theoretical multiplexing capacity of up to 500 targets, conferring a distinct advantage in multiplex pathogen detection. This platform has been successfully applied for multiplex detection in swine [15], cattle [16], and poultry viral pathogens [17,18], and also offers advantages, including enhanced specificity through dual recognition, expandable panel design, low sample volume requirements, and automated readout [19], making it particularly suitable for high-throughput routine surveillance. However, to our knowledge, no study has yet applied this technology for the simultaneous detection of all five respiratory bacterial pathogens in SPF chickens. Unlike previous Luminex assays for poultry that covered only a subset of targets or focused on viral pathogens, the present study specifically designed and optimized primer pairs for the five target pathogens, overcoming technical challenges arising from genomic differences (e.g., the GC content of *Mav* is approximately 69% [20], whereas that of *Mycoplasma* species is only 28–33% [21]).

In this study, we aimed to establish a multiplex liquid array assay based on Luminex xTAG technology for the simultaneous detection of *MG*, *MS*, *Pm*, *Apg*, and *Mav* within a single reaction system. This approach was intended to overcome the limitations of existing methods—including insufficient multiplexing capacity, procedural complexity, and incomplete coverage of the nationally required pathogens—and to provide an efficient and reliable monitoring tool for quality control in SPF chickens.

## 2. Materials and Methods

### 2.1. Bacterial Strains, DNA and Test Samples

*MG* (CVCC 352, China Veterinary Culture Collection Center, Beijing, China), *MS* (ATCC 25204, American Type Culture Collection, Manassas, VA, USA), *Apg* (ATCC 29545), and *Pm* (ATCC 43137) were preserved in our laboratory. Genomic DNA of *Mav* (CMCC 95001, China Medical Culture Collection, Beijing, China), *Mycobacterium tuberculosis* (CMCC 93009), and *Mycobacterium bovis* (CMCC 95055) were provided by the Division of Tuberculosis Vaccines and Allergen Products, National Institutes for Drug Control (NIDC, Beijing, China). A total of 18 additional non-target bacterial strains, including *Mycoplasma pneumoniae* and *Mycoplasma pulmonis*, were also stored in our laboratory for specificity testing. The complete list of these strains is provided in Section 2.9 (Specificity Test). A total of 114 nasopharyngeal swab samples were collected from SPF chickens originating from three SPF chicken breeding facilities located in Beijing and Zhejiang Province, China (one in Beijing and two in Zhejiang).

### 2.2. Design and Synthesis of Primers and Probes for qPCR

qPCR was performed to quantify *MG* DNA using primers targeting the *mgc2* gene, while *MS* DNA was quantified using qPCR with primers specific to the highly conserved *vlh2* gene, following the protocol originally reported by Raviv et al. [11] with minor modifications. For DNA quantification of other bacterial species, qPCR assays targeting the *16S rRNA* gene were conducted as described by Nadkarni et al. [22], with slight modifications (Table 1).

All qPCR reactions were set up in a total volume of 20 μL using the GoTaq^®^ Probe qPCR Master Mix (Promega, Madison, WI, USA). Each reaction mixture contained 10 μL of master mix, 0.4 μL of each 10 μM forward/reverse primer pair, 0.4 μL of probe, 6.8 μL of nuclease-free water, and 2 μL of template DNA. Amplification was performed on an ABI 7500 Fast Real-Time PCR System (Thermo Fisher Scientific, Waltham, MA, USA). The thermal cycling program was as follows: initial denaturation at 95 °C for 2 min; 40 cycles of 95 °C for 15 s and 60 °C for 60 s, with fluorescence signals recorded at the end of each extension step. All primers and probes were synthesized by Sangon Biotech (Shanghai, China).

### 2.3. Design and Synthesis of Multiplex Primers and TAG-Conjugated Probes

Specific primers for multiplex PCR were designed based on the available sequences of target genes: the *mgc2* gene of *MG* (GenBank: LS991952.1) [11], the *VY93_RS02250* gene of *MS* (GenBank: NZ_CP011096.1) [23], the *kmt1* gene of *Pm* (GenBank: CP008918.1) [24], the *hagA* gene of *Apg* (GenBank: CP050316.1) [25], and the *IS1245* gene of *Mav* (GenBank: L33879.1) [26]. Primer pairs were designed using Primer 6.0 software (PREMIER Biosoft), with the amplicon size controlled between 100 and 200 bp. The compatibility between the designed primers and the TAG sequences (complementary to the anti-TAG sequences on MagPlex-TAG microspheres) was analyzed using the Multiple Primer Analyzer tool (Thermo Fisher Scientific) to assign the appropriate microsphere numbers. A Spacer18 (iSp18) was added between the 5′ end of each forward primer and the TAG sequence, and a biotin label was attached to the 5′ end of each reverse primer [27]. After screening and validation, the final primer and TAG sequences were determined and are listed in Table 2. All primers and probes were synthesized by Sangon Biotech. The detailed primer screening and validation procedures are described in the Supplementary Materials.

### 2.4. Synthesis of Plasmid Standards

Recombinant plasmids containing the target gene fragments of *MG* (*mgc2*), *MS* (*VY93_RS02250*), *Pm* (*kmt1*), *Apg* (*hagA*), and *Mav* (*IS1245*) were constructed using the pUC57 vector. The resulting plasmids were designated as pUC57-*mgc2*, pUC57-*VY93*, pUC57-*kmt1*, pUC57-*hagA*, and pUC57-*IS1245*. All recombinant plasmids were synthesized by Sangon Biotech.

### 2.5. Genomic DNA Extraction and Quantification

Genomic DNA was extracted from target pathogens using a magnetic bead-based bacterial genomic DNA extraction kit (TIANGEN Biotech, Beijing, China) supplemented with lysozyme solution (TIANGEN) for enzymatic digestion. The extracted DNA was then quantified by qPCR using target-specific primers and probes (Table 1), and the absolute copy number was determined based on the cycle threshold (Ct) values. The genomic DNA concentration was adjusted to approximately 10^4^ copies/μL with TE buffer (Solarbio, Beijing, China) for subsequent experiments (Table 3).

### 2.6. Multiplex PCR Amplification

Genomic DNA extracted from each of the five target pathogens was used as a positive template for single-target multiplex PCR amplification. The reaction was performed in a total volume of 30 μL, containing 15 μL of 2× Multiplex PCR Plus Master Mix (QIAGEN, Hilden, Germany), 1.5 μL of each forward and reverse primer (final concentration 5 μM each), and 1 μL of template DNA. The volume was adjusted to 30 μL with nuclease-free water.

The thermal cycling program was as follows: initial denaturation at 95 °C for 5 min; 35 cycles of 95 °C for 30 s, 57 °C for 90 s, and 72 °C for 30 s; and a final extension at 68 °C for 10 min.

Amplification products were verified by agarose gel electrophoresis [28]. A 5 μL aliquot of each PCR product was mixed with 1 μL of 6× loading buffer, loaded onto a 1.0% agarose gel, and electrophoresed at a constant voltage of 100 V for 40 min in 1× TAE buffer. A DNA molecular ladder marker, positive controls (genomic DNA of the five target pathogens), and a negative control (nuclease-free water) were included in each run. Gels were visualized and documented using a gel documentation system. Subsequently, the amplified products were subjected to Sanger sequencing to confirm their identity. The sequencing results were aligned and compared against the reference sequences in the GenBank database using Snapgene (version 6.0.2) analysis.

### 2.7. Hybridization and Detection of Multiplex PCR Products

The hybridization reaction was prepared in a total volume of 100 μL, consisting of 75 μL of SAPE working solution (diluted to 10 μg/mL in 1× TMAC buffer containing 0.1% BSA), 20 μL of microsphere mix (prepared by adding 1 μL of each of the five MagPlex-TAG microspheres to 15 μL of 1× TMAC buffer), and 5 μL of the multiplex PCR product. The mixture was thoroughly vortexed and incubated at 45 °C for 30 min in a metal bath.

Detection was performed using the Luminex liquid array system with xPONENT 3.1 software (Luminex Corporation, Austin, TX, USA). Results were reported as median fluorescence intensity (MFI) for each microsphere set. MagPlex microspheres were selected as the bead type, with a sample volume of 50 μL per well and a minimum of 100 microspheres counted per target.

### 2.8. Sensitivity Test

The sensitivity of the established multiplex liquid array assay was evaluated using both single-plasmid templates and mixed-plasmid templates [29]. The five recombinant plasmid standards (pUC57-*mgc2*, pUC57-*VY93*, pUC57-*kmt1*, pUC57-*hagA*, and pUC57-*IS1245*) were separately diluted in ten-fold serial dilutions to generate six concentrations ranging from 1 × 10^5^ copies/μL to 1 copy/μL. Each dilution of the single-plasmid templates was tested individually to assess the detection capability for each target. Nuclease-free water was used as the negative control. To evaluate the performance of the multiplex method, the five plasmid standards were mixed in equal proportions and serially diluted ten-fold, yielding six concentrations with each plasmid ranging from 1 × 10^6^ copies/μL to 10 copies/μL. The mixed-plasmid dilutions were then tested using the established multiplex liquid array assay. Results were reported as MFI, and the detection sensitivity for each target was comprehensively assessed under both single-target and multiplex reaction conditions.

### 2.9. Specificity Test

Specificity testing was performed using genomic DNA from the five target pathogens and 18 non-target bacteria [30]. The non-target strains were selected based on their phylogenetic relationships: (1) closely related species (12 species) from the same genus or family, including *Mycoplasma pulmonis*, *M. pneumoniae*, *M. orale*, *M. neurolyticum*, *M. arthritidis* (same genus as *MG* and *MS*), *Pasteurella aerogenes* (same genus as *Pm*), *Avibacterium avium* and *A. gallinarum* (same genus as *Apg*), *Haemophilus haemolyticus*, *H. influenzae* (same family as *Apg*), *Mycobacterium tuberculosis*, and *M. bovis* (same genus as *Mav*); (2) *phylogenetically* related species (3 species) from the same order, including *Rodentibacter pneumotropicus*, *R. heylii*, and *Actinobacillus muris* (*Pasteurellales*); and (3) other control strains (3 species), including *Bordetella bronchiseptica*, *Streptococcus pneumoniae*, and *Streptococcus hemolyticus*. All non-target strains were tested using the established multiplex liquid array assay, and the results were interpreted based on MFI values to determine specificity for the target pathogens and the absence of cross-reactivity with non-target strains.

### 2.10. Repeatability Test

To determine the repeatability of the established multiplex method, mixed-plasmid DNA at three concentration levels—1 × 10^6^ copies/μL, 1 × 10^4^ copies/μL, and 1 × 10^2^ copies/μL—was used as the template, with nuclease-free water as the negative control. Both intra- and inter-assay repeatability were assessed by performing three replicate tests within the same run and on three different days, respectively. The stability and repeatability of the method were evaluated by calculating the coefficient of variation (CV) of the MFI values for each concentration.

### 2.11. Detection of Simulated Positive Samples

Genomic DNA of the five target pathogens, extracted and quantified as described in Section 2.6, was used as simulated positive samples. These DNA templates were mixed in various combinations to generate 16 different mixtures [31], covering single infections, dual infections, triple infections, four-plex infections, and five-plex infections (specific combinations are detailed in Table 4). Each mixed sample was tested using the established multiplex liquid array assay following the amplification and hybridization procedures described above. Results were interpreted based on MFI values to evaluate the capability of the method to correctly identify single and mixed infections.

### 2.12. Sample Detection

To evaluate the practical applicability of the established method, genomic DNA extracted from 114 SPF chicken nasopharyngeal swab samples (as described in Section 2.5) was tested using the developed multiplex liquid array assay. Positive controls (a mixture of genomic DNA from the five target pathogens) and negative controls (nuclease-free water) were included in each run. Multiplex PCR amplification and liquid array hybridization were performed under the optimized conditions described above, and results were interpreted based on MFI values. For comparison, the same set of swab samples was also tested using a previously reported real-time PCR methods [11,32,33,34], and the results were compared with those obtained by the developed method.

### 2.13. Verification of Primer and Platform Adaptability

Three independent verification experiments were performed using the established multiplex liquid array assay to assess the adaptability of previously reported primers on the Luminex platform and their compatibility with the primers designed in this study. Detailed primer sequences are provided in the Supplementary Materials (Table S1).

Briefly, previously reported qPCR primers for *MG*, *Pm*, and *Apg* [10] were modified according to the primer design strategy of the present study (i.e., addition of an iSp18 and TAG sequence to the 5′ end of each forward primer, and biotin labeling at the 5′ end of each reverse primer), and tested in a triplex format on the Luminex platform. Subsequently, these modified primers were combined with the *MS* and *Mav* primers designed in this study to assess their compatibility in a multiplex system. In addition, previously reported liquid array primers for *MG* and *MS* from the Guangdong Provincial Local Standard DB44/T 2338-2021 [35] were modified by replacing their original TAG sequences with those used in this study, and tested in combination with the *Pm*, *Apg*, and *Mav* primers of the present study. Finally, an alternative pair of *Mav* primers (Mav-F2/R2) was designed based on the *IS1245* gene sequence to replace the original *Mav* primer pair, and its performance in the multiplex system was evaluated. All tests were performed using ten-fold serially diluted mixtures of genomic DNA from the five target pathogens.

### 2.14. Statistical Analysis

For analytical validation experiments (sensitivity, specificity, and repeatability), all tests were performed in at least three independent replicates. For repeatability assessment, data were expressed as mean ± standard deviation (SD) of MFI values, and the CV was calculated as (SD/mean) × 100, with a CV below 15% considered acceptable. Intra-assay repeatability was evaluated by testing three replicates within the same run, while inter-assay repeatability was assessed by performing the assay on three different days. For sample positivity determination, a sample was considered positive when the MFI value of the corresponding microsphere set was ≥3-fold that of the negative control and simultaneously >300; otherwise, it was considered negative. For simulated positive samples, descriptive comparison was used to evaluate concordance between expected and observed results. For clinical sample detection (*n* = 114), Cohen’s kappa coefficient (κ) and overall agreement percentage were calculated to compare the multiplex method with qPCR results. All statistical analyses were performed using GraphPad Prism 8.0 software (GraphPad Software, San Diego, CA, USA) and WPS Excel (version 12.1, Kingsoft Office).

## 3. Results

### 3.1. Multiplex PCR Amplification Results

**Figure 1:**
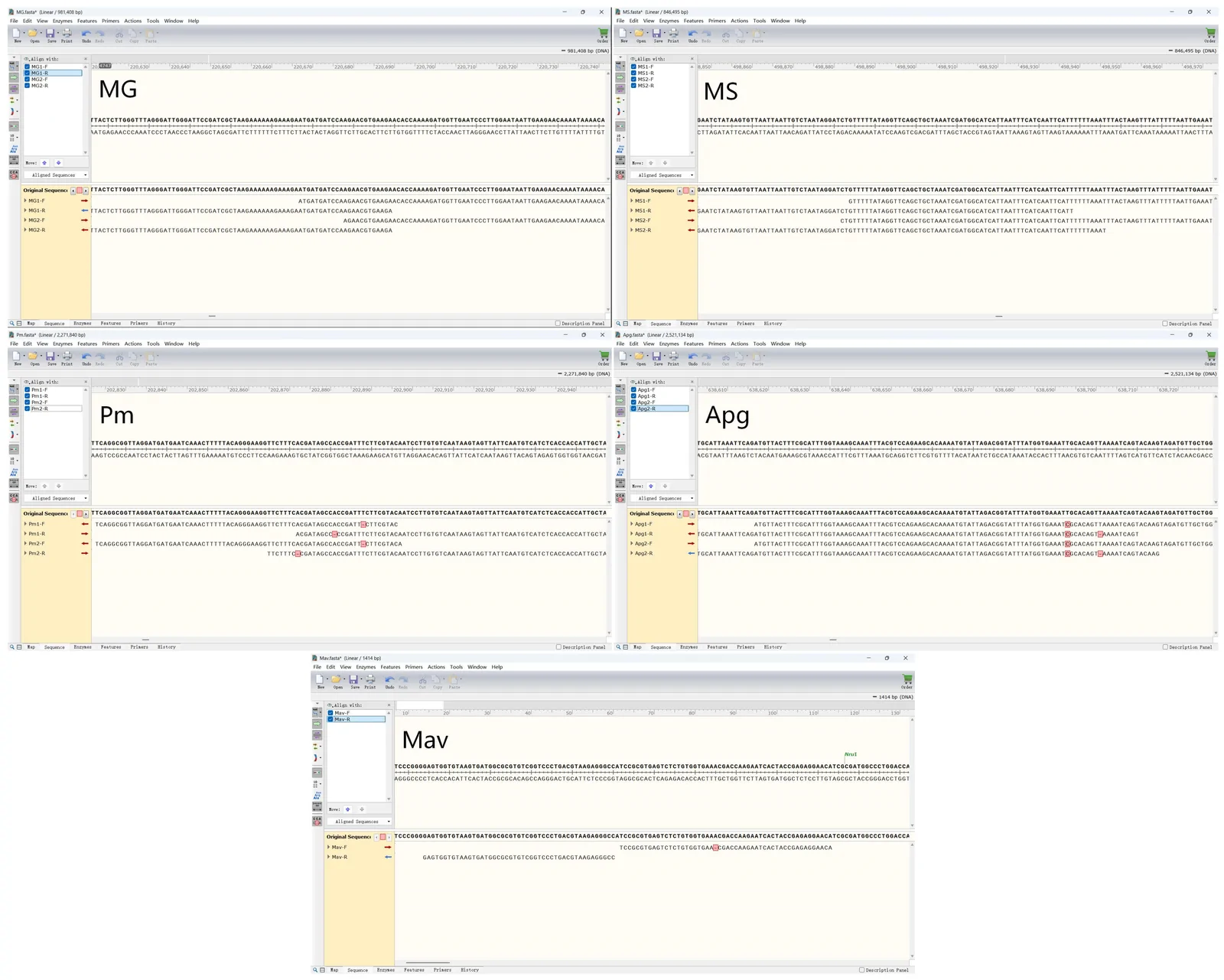
Sanger sequencing confirmation of amplified products. Representative sequence alignments showing >98% identity of the amplified products to the reference sequences of the corresponding target genes for each pathogen.

Multiplex PCR amplification was performed using genomic DNA of the five target pathogens as templates, and the products were analyzed by 1% agarose gel electrophoresis. The results showed specific bands at the expected sizes for each target, with no non-specific amplification observed, and no bands were detected in the negative control. To confirm that the amplified fragments corresponded to the correct target sequences, the PCR products from the standard strains were sequenced. The sequencing alignment results confirmed that the amplified fragments from each primer pair showed >98% sequence identity with the reference sequences of the corresponding target genes, with the observed minor nucleotide variations being within the expected range of natural genetic diversity. The sequencing alignment results are presented in Figure 1, while the original gel images are available in the Supplementary Materials (Figure S1). These results demonstrate that the designed primers exhibit good amplification specificity in the multiplex PCR system.

### 3.2. Sensitivity Tests Results

The sensitivity of the established multiplex liquid array assay was evaluated using both single-plasmid and mixed-plasmid templates. The five recombinant plasmid standards were serially diluted ten-fold to concentrations ranging from 1 × 10^5^ copies/μL to 1 copy/μL for individual template testing. Additionally, the five plasmids were mixed in equal proportions and serially diluted ten-fold to concentrations ranging from 1 × 10^6^ copies/μL to 10 copies/μL for mixed template testing. Nuclease-free water was used as the negative control, and results were interpreted based on MFI values.

**Figure 2:**
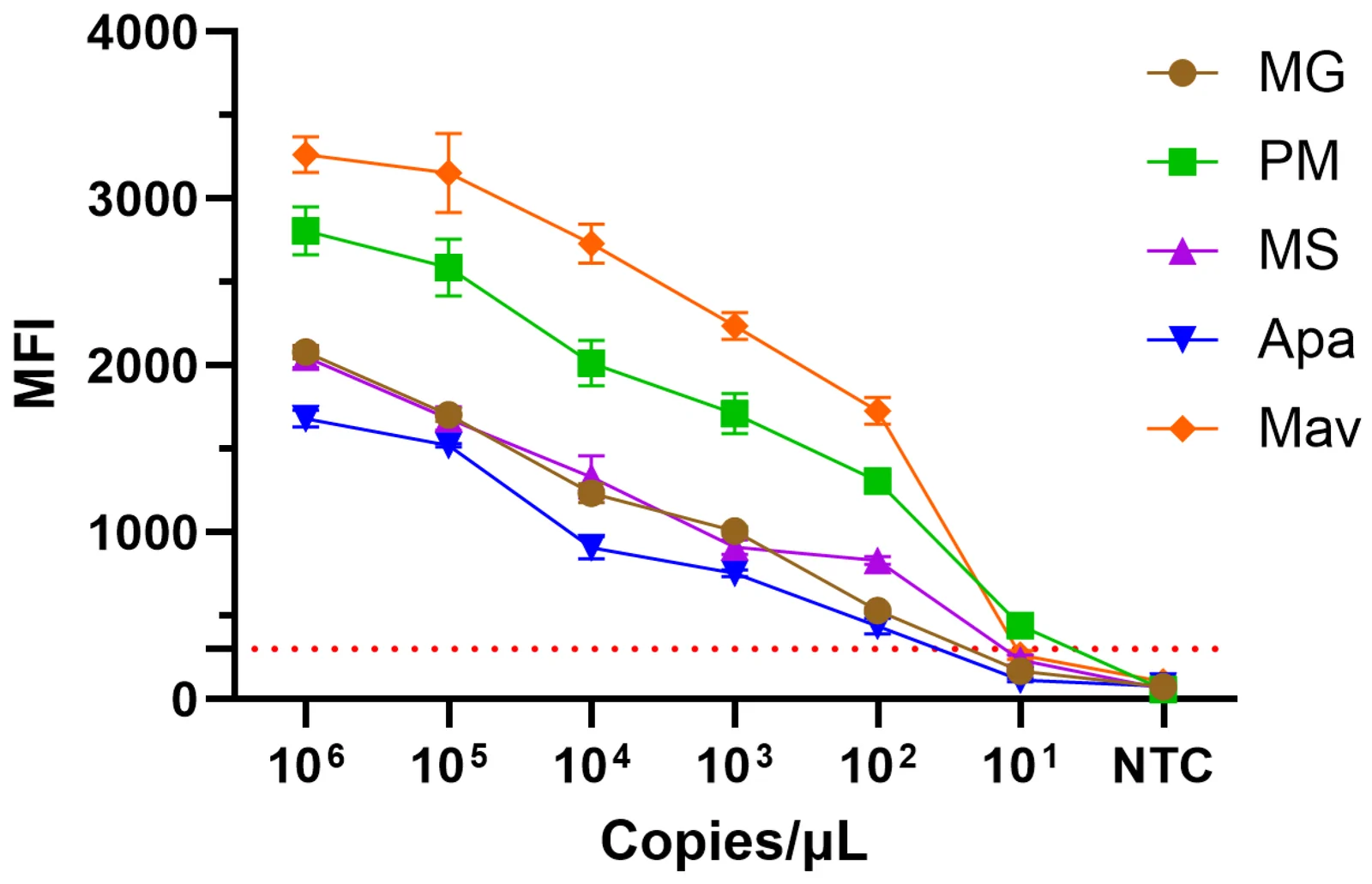
Sensitivity test Results (single plasmids, MFI values), the red dotted line represents the approximate positive cutoff line at 300 MFI.

For single-template detection, the limit of detection (LOD) for all five targets was 10 copies/μL (Figure 2). For mixed-template detection, the LOD for *Pm* reached 10 copies/μL (MFI = 437), while the LODs for *MG*, *MS*, *Apg*, and *Mav* were 100 copies/μL each (Table 5).

### 3.3. Specificity Test Results

**Figure 3:**
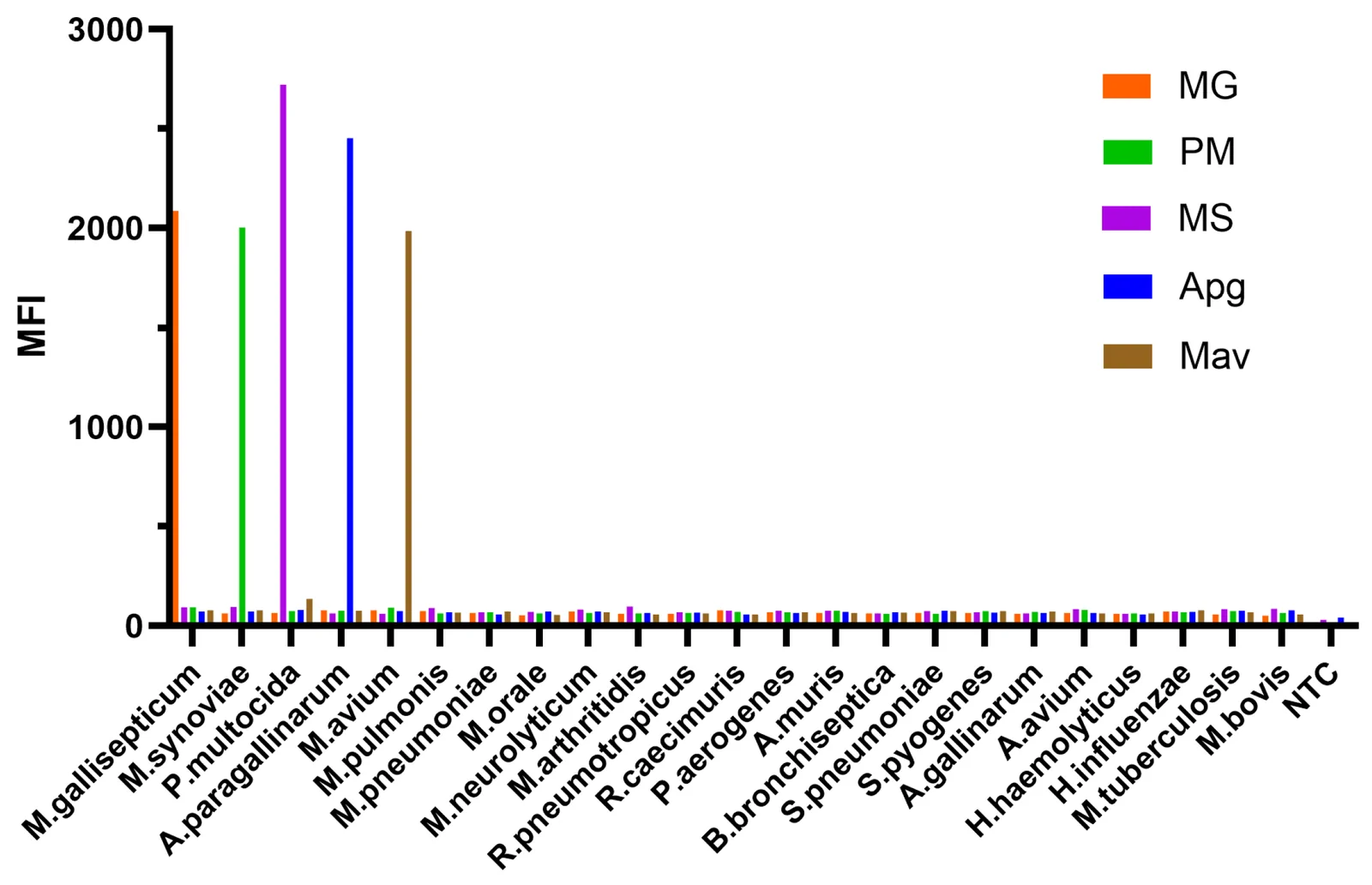
Specificity test results of the multiplex liquid array assay for the five target pathogens and 18 non-target bacterial species. MFI values for the five target pathogens (*MG*, *MS*, *Pm*, *Apg*, and *Mav*) were significantly higher than those of the negative controls, while all non-target species tested negative, demonstrating no cross-reactivity.

The specificity of the established method was evaluated by testing genomic DNA from the five target pathogens and 18 non-target bacterial species. As shown in Figure 3, the MFI values for the five target pathogens were significantly higher than those of the negative controls: *MG*, 2085.5; *MS*, 2003; *Pm*, 2721; *Apg*, 2452.5; and *Mav*, 1985. In contrast, all non-target bacterial species tested negative, with no significant difference from the negative control. These results demonstrate that the developed assay exhibits excellent specificity for the five target pathogens, with no cross-reactivity observed against the 18 non-target species tested, including *Mycoplasma pneumoniae*, *Mycoplasma pulmonis*, *Mycobacterium tuberculosis*, and *Mycobacterium bovis*. The detailed MFI values for all tested strains are provided in Table S2 in the Supplementary Materials.

### 3.4. Repeatability Test Results

Intra-assay and inter-assay repeatability were evaluated using mixed plasmid templates at three concentration levels: 1 × 10^6^ copies/μL, 1 × 10^4^ copies/μL, and 1 × 10^2^ copies/μL. As shown in Table 6, the CVs for all targets at all concentrations were below 15%, with intra-assay CV values ranging from 0.8% to 12.2% and inter-assay CV values ranging from 2.3% to 14.4%. These results indicate that the developed method exhibits excellent repeatability and stability.

### 3.5. Detection Results of Simulated Positive Samples

**Figure 4:**
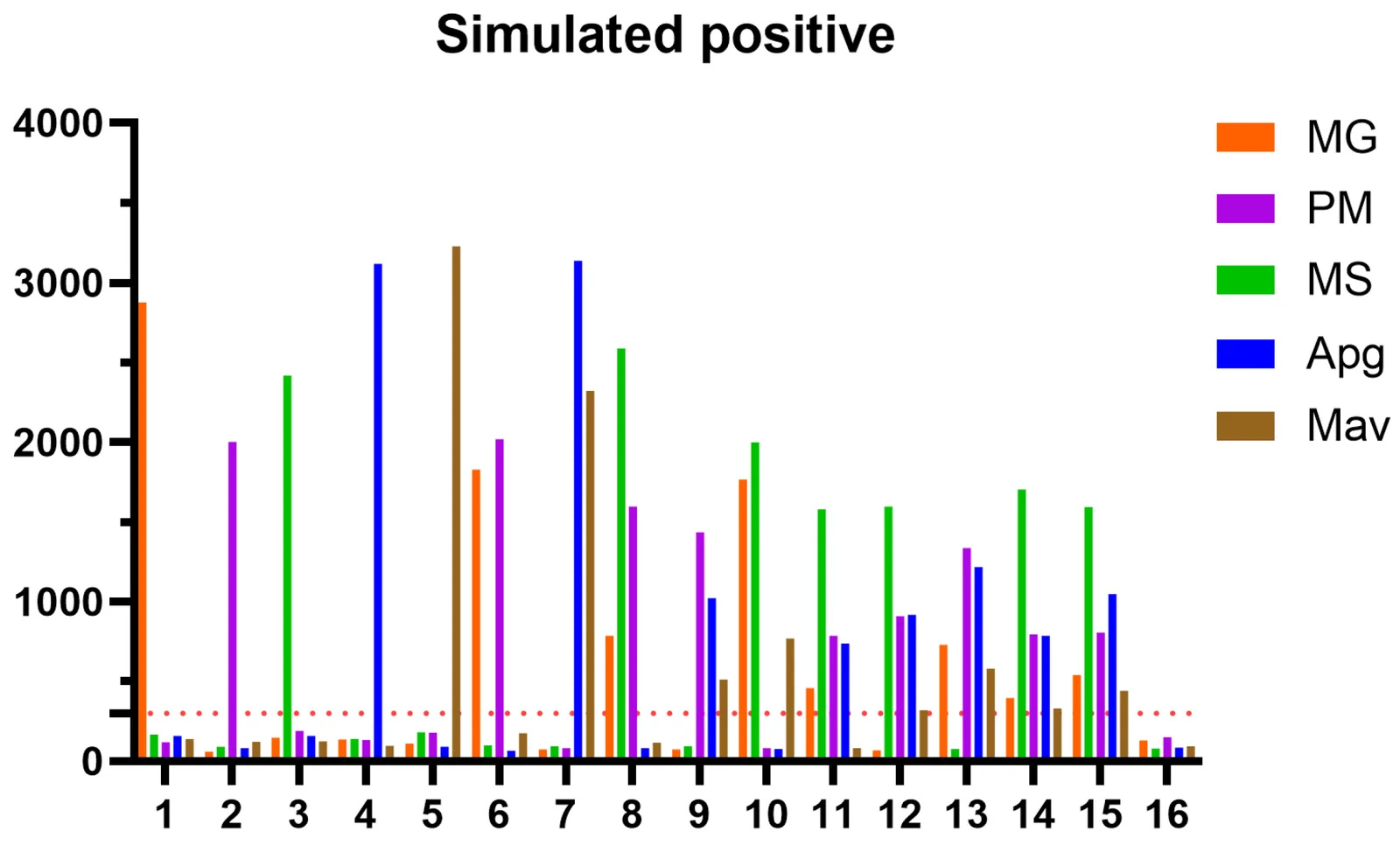
Results of Simulated Positive Samples. Each bar denotes the MFI of simulated positive samples, and the red dotted line represents the approximate positive cutoff line at 300 MFI.

Simulated detection was performed using 16 different combinations of the five pathogen genomic DNA templates, ranging from single to five-plex infections. As shown in Table 7, the detection results for all combinations were completely consistent with the expected combinations. The MFI values for all positive targets were significantly higher than those of the negative controls (≥300 and ≥3-fold that of the negative control MFI), while the MFI values for all negative targets remained below the threshold. Notably, as the number of mixed targets increased, the MFI values of some targets showed varying degrees of reduction; however, these reductions did not affect the final qualitative result. These results demonstrate that the developed method can accurately identify both single and mixed infections in samples (Figure 4).

### 3.6. Sample Detection Results

The developed method was applied to detect genomic DNA extracted from 114 SPF chicken nasopharyngeal swab samples. All samples tested negative based on the MFI threshold criteria, while the positive controls were valid, indicating that the method can be effectively used for routine microbiological monitoring of SPF chickens (partial MFI values are provided in the Table S3). In comparison, qPCR detection using the previously reported method revealed a positive rate of 20.2% (23/114) for *MG*, with Ct values of 35.53 ± 0.85. Based on the standard curve equation, the nucleic acid concentration in the positive samples was calculated to be 1.3 ± 0.3 copies/μL. The other four target pathogens were also negative by qPCR, with no corresponding nucleic acids detected (Table 8).

### 3.7. Verification of Primer and Platform Adaptability Results

To evaluate the adaptability of previously reported primers on the Luminex platform and their compatibility with the primers designed in this study, three independent verification experiments were conducted. Detailed results, including MFI values for each microsphere set, are provided in the Supplementary Materials (Tables S4–S7).

The previously reported qPCR primers for *MG*, *Pm*, and *Apg* [10] were modified and tested on the Luminex platform. However, severe non-specific background signals and cross-reactivity were observed in the negative controls, indicating that these primers cannot be directly adapted to the Luminex platform simply by adding TAG sequences (Table S4).

When the modified qPCR primers were combined with the *MS* and *Mav* primers of the present study, high background signals persisted for *Pm* and *Mav* in the negative controls, resulting in false-positive results and demonstrating poor compatibility between the two primer sets (Table S5).

Similarly, previously reported liquid array primers for *MG* and *MS* from the Guangdong Provincial Local Standard DB44/T 2338-2021 [35], when modified and combined with the *Pm*, *Apg*, and *Mav* primers of the present study, exhibited significant non-specific signal elevation in the negative controls, leading to false-positive results and failure to construct a functional multiplex system (Table S6).

Finally, an alternative *Mav* primer pair (Mav-F2/R2) designed based on the *IS1245* gene sequence was tested in combination with the other four primer pairs. Abnormally elevated MFI values were observed for multiple targets in the negative controls, with particularly high non-specific signals for *Mav*, rendering the alternative primers unsuitable for practical detection applications (Table S7).

Collectively, these results confirm that previously reported primers—whether originally designed for qPCR or liquid array platforms—cannot be effectively adapted to the Luminex-based multiplex liquid array assay of the present study through simple modification, underscoring the specificity and robustness of the primer sets developed in this work.

## 4. Discussion

In this study, a multiplex liquid array nucleic acid detection method based on Luminex xTAG technology was successfully established, enabling the simultaneous detection of all five SPF chicken respiratory bacterial pathogens mandated by the Chinese national standard GB/T 17999.1-2008 [1] and the Chinese Pharmacopoeia (2025 Edition) [2] in a single reaction system. This method fills the application gap of Luminex xTAG technology for the simultaneous detection of these five pathogens in SPF chickens, providing a comprehensive technical solution for SPF chicken quality control.

The Luminex xTAG liquid array technology, based on fluorescently coded microspheres and flow-based fluorescent detection, offers a theoretical multiplexing capacity of up to 500 targets [36]. Compared with traditional detection methods, this technology demonstrates significant advantages not only in multiplexing capability but also in detection throughput, sample consumption, and automation [19]. The multiplex liquid array assay established in this study fully exploits these technological features, integrating the five respiratory bacterial pathogens mandated by Chinese national standards into a single reaction system, thereby overcoming the limitation of qPCR methods that are typically restricted to 4–6 targets per reaction due to fluorescence channel constraints. Notably, Wang et al. [10] recently reported a four-plex qPCR method for quails enabling simultaneous detection of *Pm*, *Apg*, *MG*, and *MS*; however, their target panel still did not include *Mav*, which may be constrained by the multiplexing capacity of the qPCR platform. The present study further expands the target coverage on this basis, demonstrating the unique advantages of the Luminex platform in multiplex detection.

The sensitivity results showed that *Pm* could be detected at 10 copies/μL, while the other four pathogens had a LOD of 100 copies/μL, which meets the requirements for routine surveillance of SPF chickens [37,38]. The repeatability results showed that both intra- and inter-assay CV were below 15%, indicating good stability and reproducibility of the method. In the specificity tests, none of the 18 non-target bacterial species showed cross-reactivity, confirming the rigor of the primer design and the high specificity of the method. Notably, the primers for *MG* and *MS* target the *mgc2* and *VY93_RS02250* genes [39,40,41], respectively, which have been previously validated for their species-specificity; similarly, the primers for *Pm* and *Apg* target the *kmt1* and *hagA* genes [42,43,44], respectively, both of which are conserved specific regions validated in the literature.

The primer screening and optimization process in this study provides important methodological insights. Verification experiments demonstrated that directly “transplanting” previously reported qPCR primers or liquid array primers from existing standards into the Luminex system of the present study—by simply adding TAG sequences and biotin labels—resulted in severe non-specific background signals and false-positive outcomes. This phenomenon suggests that different detection platforms (qPCR vs. liquid array) impose substantially different performance requirements on primers, and simple primer modification does not guarantee platform compatibility. This further underscores the necessity of the substantial effort invested in de novo primer design and systematic screening in the present study. The hybridization reaction conditions, microsphere surface chemistry, and signal readout mechanisms of the Luminex platform are fundamentally different from those of qPCR, requiring that platform-specific factors be taken into consideration during primer design, rather than simply adopting existing primer sequences.

The five target pathogens exhibit significant differences in genomic characteristics, which poses additional challenges for establishing a unified multiplex method. The GC content of *Mav* is approximately 69% [20,45], whereas that of *Mycoplasma* species is only 28–33% [21]. This substantial difference in genomic composition directly affects the coordination of primer annealing temperatures and the balance of amplification efficiency. Through strict control of amplicon lengths (101–191 bp) and optimization of the annealing temperature (57 °C), the primer design in this study successfully achieved balanced amplification of all five pathogens in a single reaction system, providing a referable strategy for handling mixed targets with markedly different genomic features.

From the perspective of practical application, the method established in this study has undergone preliminary validation with 114 nasopharyngeal swab samples from SPF chickens, all of which tested negative, consistent with the expected microbiological status of SPF chickens. Mixed infections with avian respiratory pathogens are extremely common in clinical settings, and such co-infections often lead to more severe clinical manifestations than single infections [46]. The flexible panel design of the Luminex platform allows for the addition or removal of targets as monitoring needs evolve, providing a technical foundation for future expansion of this method to include simultaneous detection of more SPF chicken pathogens, such as viral pathogens. Furthermore, MG and MS are vertically transmitted through eggs, making their exclusion status particularly critical for maintaining SPF chicken quality [8,47]. The high sensitivity and specificity of this method enable accurate detection at early stages of infection, which is of great significance for the prevention and control of vertically transmitted pathogens.

However, several limitations of this method need to be clearly acknowledged. In comparative sensitivity testing, the LOD of the multiplex liquid array assay for MG was 100 copies/μL, which is higher than that reported for single-plex quantitative PCR (approximately 1 copy/μL). When validating with 114 SPF chicken samples, qPCR detected 23 MG-positive samples with a Ct value of 35.53 ± 0.85, corresponding to approximately 1.3 ± 0.3 copies/μL; in contrast, the present method yielded negative results for all samples, indicating inferior detection capability for samples with extremely low bacterial loads compared to qPCR. This is likely attributable to the fact that the Luminex platform requires a two-step procedure (multiplex PCR amplification followed by hybridization reaction), and its signal accumulation efficiency is lower than that of single-plex qPCR. Nevertheless, it must be clearly emphasized that although the sensitivity of this method is slightly lower than that of single-target qPCR, the LOD of 100 copies/μL is fully adequate for routine monitoring of SPF chickens—because the core objective of SPF quality control is to exclude the presence of target pathogens, rather than to pursue the absolute lowest detection threshold. This LOD is consistent with industry-recognized standards: commercial *MG* detection kits typically define Ct ≤ 35 as positive (*MG* Nucleic Acid Detection Kit, PCR-Fluorescence Probe Method, TSTOV0096), and some international brands claiming LODs of 500 copies/μL (e.g., InviScreen^®^ *Mycoplasma* (*Mg*/*Ms)* Detection Kit, No.6016003200), and published qPCR studies have reported LODs of 10^2^–10^1^ copies/μL from artificially contaminated swab samples [48]. In practical applications, the bacterial loads in naturally infected cases typically range from 10^3^ to 10^8^ copies/μL [37,49], far exceeding the LOD of the present method. Therefore, from a practical application perspective, this method, characterized by high throughput, cost-effectiveness, and multiplexing capability, offers greater value for routine monitoring than the pursuit of extremely high sensitivity; these advantages are particularly noteworthy in large-scale SPF chicken quality monitoring programs. In addition, the risk of missing samples with extremely low loads could be further reduced by employing magnetic bead-based nucleic acid pre-concentration or pre-enrichment.

Furthermore, it is necessary to clarify the complementary role of this method relative to qPCR: the present assay is specifically designed for routine monitoring as a high-throughput screening tool, whereas qPCR is more suitable for confirmatory testing of samples with ambiguous signals or low target levels. This complementary positioning provides a clear basis for the rational selection of the two methods in practical applications. Although the current detection panel covers five major respiratory bacterial pathogens, future expansion may be needed to address emerging pathogens or evolving testing requirements; at the same time, the long-term applicability of this method for large-scale sample testing in routine production environments and different poultry farm settings still requires further validation through continuous data accumulation. It should be particularly noted that all samples tested in this study were derived from SPF chickens and were negative for all five target pathogens; therefore, the diagnostic performance of this method in naturally infected or suspected samples has not yet been fully validated. As a preliminary study focused on method establishment and analytical validation, the present results have demonstrated the technical feasibility of this detection method for SPF chicken quality monitoring. Future evaluation using commercial chicken flocks or outbreak field samples is needed to comprehensively determine its sensitivity and specificity.

## 5. Conclusions

In this study, a multiplex liquid array detection method based on Luminex xTAG technology was successfully established. This method enables the simultaneous detection of all five SPF chicken respiratory bacterial pathogens mandated by the Chinese national standard GB/T 17999.1-2008 [1] and the Chinese Pharmacopoeia (2025 Edition) [2]—*MG*, *MS*, *Pm*, *Apg*, and *Mav*—in a single reaction system. The developed assay offers several advantages, including high throughput, high sensitivity (10 copies/μL for Pm and 100 copies/μL for the other four targets), high specificity (no cross-reactivity with 18 non-target bacterial species), and good repeatability (CV < 15%). The detection results for both simulated and routine samples were consistent with expectations, confirming the reliability and practical applicability of the method. Collectively, this method provides an efficient and convenient monitoring tool for SPF chicken quality control, with significant potential for broad application and translation (Table S8).

## 6. Patents

A patent application has been submitted for this research under application number 202610846363X.

## Figures and Tables

**Table 1:** Primers and probes used for qPCR quantification.

Pathogen	Target Gene	Primer	Sequence
*MG*	*mgc2*	mgc2-F	TTGGGTTTAGGGATTGGGATT
		mgc2-R	CCAAGGGATTCAACCATCTT
		mgc2-P	FAM-TGATGATCCAAGAACGTGAAGAACACC-BHQ1
*MS*	*vlhA*	VlhA-F	ATAGCAATTTCATGTGGTGATCAA
		VlhA-R	TGGATTTGGGTTTTGAGGATTA
		VlhA-P	ROX-CAGCACCTGAACCAACACCTGGAA-Eclipse
Other bacteria	*16S rRNA*	16S-F	TCCTACGGGAGGCAGCAGT
		16S-R	GGACTACCAGGGTATCTAATCCTGTT
		16S-P	FAM-CGTATTACCGCGGCTGCTGGCAC-BHQ1

**Table 2:** Primers and TAG sequences for the multiplex liquid array detection of five SPF chicken respiratory bacterial pathogens.

Primer	Target Gene	Accession Number	Location	Sequence	Product Size (bp)	MagPlex-TAG™ Microsphere Part Numbers
MG-F	*mgc2*	LS991952.1	220613~220633	CAAATACATAATCTTACATTCACT/iSp18/TCTTTACTCTTGGGTTTAGGG	143	13
MG-R			220738~220755	5′Biotin-GGCTCAATCGCTTCTGTT		
MS-F	*VY93_RS02250*	CP011096.1	498839~498859	ATACTTTACAAACAAATAACACAC/iSp18/GGCGAGTTGAATCTATAAGTG	155	19
MS-R			498974~498993	5′Biotin-TCTGCTTTAAGAAATGCTGC		
PM-F	*kmt1*	CP008918.1	202929~202946	TACTTCTTTACTACAATTTACAAC/iSp18/TAGCAATGGTGGTGAGATG	124	15
PM-R			202824~202841	5′Biotin-CAGGCGGTTAGGATGATG		
Apg-F	*hagA*	CP050316.1	638567~638585	AATCTCTACAATTTCTCTCTAATA/iSp18/CACCAGTTGTTGAACCTAAG	191	61
Apg-R			638739~638756	5′Biotin-TGCTTCGCTACCAATACG		
Mav-F	*IS1245*	L33879.1	13~31	CTAAACATACAAATACACATTTCA/iSp18/GGAGTGGTGTAAGTGATGG	101	62
Mav-R			95~113	5′Biotin-TGTTCCTCTCGGTAGTGAT		

**Table 3:** Concentrations of genomic DNA from the five target pathogens used in this study.

Pathogen	Copy Number (Copies/μL)	DNA Concentration (ng/μL)	Working Concentration for Simulated Positive Samples (Copies/μL)
*MG*	2.02 × 10^8^	217	2.02 × 10^4^
*MS*	2.02 × 10^6^	1.9	2.02 × 10^4^
*Pm*	1.11 × 10^7^	28	1.11 × 10^4^
*Apg*	9.32 × 10^7^	255	9.32 × 10^4^
*Mav*	6.57 × 10^4^	3.6	6.57 × 10^4^

**Table 4:** Different combinations of the five target pathogen DNA.

No.	Combinations	No.	Combinations
1	*MG*	9	*MS + Apg + Mav*
2	*MS*	10	*MG + Pm + Mav*
3	*Pm*	11	*MG + MS + Pm + Apg*
4	*Apg*	12	*MS + Pm + Apg + Mav*
5	*Mav*	13	*MG + MS + Apg + Mav*
6	*MG + MS*	14	*MG + MS + Pm + Apg + Mav*
7	*Apg + Mav*	15	*MG + MS + Pm + Apg + Mav*
8	*MG + MS + Pm*	16	NC

**Table 5:** Sensitivity test Results (mixed plasmids, MFI values).

Plasmid Concentration (Ccopies/μL)	*MG*		*PM*		*MS*		*Apg*		*Mav*	
	Mean MFI	SD	Mean MFI	SD	Mean MFI	SD	Mean MFI	SD	Mean MFI	SD
10^6^	2081	39	2809	144	2051	64	1681	51	3266	108
10^5^	1704	40	2590	171	1682	71	1522	10	3157	239
10^4^	1235	56	2014	138	1332	127	908	67	2730	118
10^3^	1007	29	1713	119	910	45	756	23	2240	80
10^2^	530	16	1309	33	833	24	437	47	1727	82
10	168	10	442	38	236	31	116	11	265	27
NTC	76	11	58	7	62	5	79	10	105	10

**Table 6:** Intra- and inter-assay repeatability test results of the multiplex method.

Target	Plasmid Concentration (Copies/μL)	Intra-Assay			Inter-Assay		
		Mean	SD	CV(%)	Mean	SD	CV(%)
*MG* (pUC57-mgc2)	10^6^	1966.8	119.0	6.0	2010.5	87.0	4.3
	10^4^	1437.8	11.4	0.8	1581.3	36.3	2.3
	10^2^	546.0	34.4	6.3	522.5	54.3	10.4
*MS* (pUC57-VY93_RS02250)	10^6^	1401.2	120.0	8.6	1673.3	80.2	4.8
	10^4^	906.7	16.3	2.1	1060.5	138.4	13.0
	10^2^	401.3	13.7	3.4	421.0	41.2	9.8
*Pm* (pUC57-kmt1)	10^6^	2170.7	119.2	5.5	2334.2	263.3	11.3
	10^4^	1516.7	127.0	8.4	1499.7	169.4	11.3
	10^2^	851.2	72.5	8.5	702.2	101.3	14.4
*Apg* (pUC57-hagA)	10^6^	1474.2	154.5	10.5	1725.2	53.5	3.1
	10^4^	928.0	15.9	1.7	994.7	129.5	13.0
	10^2^	528.0	15.6	3.0	436.7	23.4	5.4
*Mav* (pUC57-IS1245)	10^6^	3367.5	411.8	12.2	3771.8	313.3	8.3
	10^4^	2592.8	73.1	2.8	2513.2	75.9	3.0
	10^2^	1302.8	81.8	6.3	1401.0	60.3	4.3

**Table 7:** Results of the multiplex simulation test (partial, MFI values).

No.	Combinations	No. Microsphere and MFI Values				
		No. 13	No. 15	No. 19	No. 61	No. 62
1	*MG*	2876.0	169.0	120.5.0	158.5	139.0
2	*MS*	61.0	92.5	2003.0	84.0	122.0
3	*Pm*	148.5	2419.0	191.0	159.5	126.5
4	*Apg*	138.0	139.0	134.0	3118.0	96.5
5	*Mav*	110.5	181.0	178.0	92.5	3228.0
6	*MG + MS*	1829.0	101.0	2020.0	66.0	177.0
7	*Apg + Mav*	75.5	95.0	84.0	3138.0	2321.0
8	*MG + MS + Pm*	787.0	2586.0	1597.5	83.0	118.0
9	*MS + Apg + Mav*	74.0	95.0	1436.0	1023.0	512.5
10	*MG + Pm + Mav*	1767.0	2001.0	83.0	77.0	770.0
11	*MG + MS + Pm + Apg*	457.5	1579.0	785.0	738.0	84.0
12	*MS + Pm + Apg + Mav*	70.5	1597.5	909.0	918.0	320.0
13	*MG + MS + Apg + Mav*	729.5	78.0	1338.0	1217.0	581.0
14	*MG + MS + Pm + Apg + Mav*	398.0	1702.5	796.0	785.0	332.5
15	*MG + MS + Pm + Apg + Mav*	539.5	1594.0	807.0	1049.0	443.0
16	NTC	130.0	81.5	152.0	85.5	93.5

**Table 8:** Comparison of detection results between the developed multiplex liquid array assay and qPCR for 114 SPF chicken nasopharyngeal swab samples (*n* = 114).

Pathogen	Multiplex Method (Positive/Total)	qPCR (Positive/Total)	Overall Agreement (%)	Cohen’s Kappa (κ)	95% CI
*MG*	0/114	23/114	79.8%	0.000	−0.102–0.102
*MS*	0/114	0/114	100%	N.D.	N.D.
*Pm*	0/114	0/114	100%	N.D.	N.D.
*Apg*	0/114	0/114	100%	N.D.	N.D.
*Mav*	0/114	0/114	100%	N.D.	N.D.

Note: N.D. = not defined. For targets where both methods yielded all-negative results (*MS*, *Pm*, *Apg*, and *Mav*), Cohen’s kappa coefficient and its 95% CI were not computed due to the absence of discordant events (where P_0_ = P_e_ = 1, resulting in a 0/0 indeterminate form). In such cases, complete concordance was determined based on the 100% overall agreement rate. For *MG*, where discordance was observed, Cohen’s kappa was calculated using the standard asymptotic method, yielding κ = 0.000 (95% CI: −0.102–0.102); this result indicates that the agreement between the two methods is no better than chance, and the observed discordance is attributable to the fact that the LOD the multiplex liquid array assay (100 copies/μL) is higher than the bacterial load of the qPCR-positive samples (1.3 ± 0.3 copies/μL).

## Data Availability

The original contributions presented in this study are included in the article. Further inquiries can be directed to the corresponding author.

## Supplementary Materials

The following supporting information can be downloaded at: https://www.mdpi.com/article/10.3390/vetsci13080753/s1, Text: the detailed primer screening and validation procedures, Table S1: primer sequences used for adaptability verification, Figure S1: original gel electrophoresis images, Table S2: specificity test results, Tables S3–S7: primer and platform adaptability verification data, and Table S8: a summary of analytical performance metrics.

## References

[1] (2008). SPF Chicken—Microbiological Surveillance—Part 1: General Rules for the Microbiological Surveillance for SPF Chicken.

[2] Chinese Pharmacopoeia Commission (2025). Pharmacopoeia of the People’s Republic of China, Volume IV—General Chapter 3601.

[3] Miller J.M., Ozyck R.G., Pagano P.L., Hernandez E.F., Davis M.E., Karam A.Q., Malek J.B., Mara A.B., Tulman E.R., Szczepanek S.M. (2024). Rationally designed *Mycoplasma gallisepticum* vaccine using a recombinant subunit approach. npj Vaccines.

[4] El-Gazzar M. (2025). *Mycoplasma synoviae* infection in poultry. Merck Veterinary Manual.

[5] Lighty M. (2024). Fowl cholera. Merck Veterinary Manual.

[6] Wang Y., Si M., Qiu J., Chong X., Yang B., Chu M., Liang Y., Cheng W., Zhang H., Chen X. (2026). Prevalence, risk factors, and regional insights of infectious coryza among poultry populations in China during 1993–2024: A meta-analysis. J. Vet. Med. Sci..

[7] Puspitasari Y., Khairullah A.R., Raharjo H.M., Tyasningsih W., Kurniasih D.A.A., Moses I.B., Wardhani B.W.K., Ekawasti F., Pratama B.P., Rehman S. (2026). Current perspectives on avian tuberculosis in domestic and wild birds: Molecular diagnostics, genomic surveillance, and vaccination challenges. Open Vet. J..

[8] Yadav J.P., Tomar P., Singh Y., Khurana S.K. (2022). Insights on *Mycoplasma gallisepticum* and *Mycoplasma synoviae* infection in poultry: A systematic review. Anim. Biotechnol..

[9] Huang Q., Yang X., Zhao X., Han X., Sun S., Xu C., Cui N., Lu M. (2024). Co-infection of H9N2 subtype avian influenza virus and QX genotype live attenuated infectious bronchitis virus increase the pathogenicity in SPF chickens. Vet. Microbiol..

[10] Wang H., Xue L., Wang L., Liu Y., Chen J., Sun Y., An T., Li C., Chen H., Yu C. (2025). The quadruplex fluorescent quantitative PCR method for the simultaneous detection of respiratory diseases in quail: *Pasteurella multocida*, *Avibacterium paragallinarum*, *Mycoplasma gallisepticum*, and *Mycoplasma synoviae*. Front. Microbiol..

[11] Raviv Z., Kleven S.H. (2009). The development of diagnostic real-time TaqMan PCRs for the four pathogenic avian mycoplasmas. Avian Dis..

[12] Xu B., Wang S., Yao W., Ni B., Yuan T., Liu B., Yuan L., Wei Y., Ma S., Lyu L. (2025). New molecular diagnostic targets for *Avibacterium paragallinarum* and a set of single-plex and multiplex qPCR methods for the rapid differential diagnosis of *Mycoplasma gallisepticum*, *Mycoplasma synoviae*, and *Avibacterium paragallinarum*. Poult. Sci..

[13] Wei B., Cha S.Y., Kang M., Park I.-J., Moon O.-K., Park C.-K., Jang H.-K. (2013). Development and application of a multiplex PCR assay for rapid detection of 4 major bacterial pathogens in ducks. Poult. Sci..

[14] Gopinath K., Singh S. (2009). Multiplex PCR assay for simultaneous detection and differentiation of *Mycobacterium tuberculosis*, *Mycobacterium avium* complexes and other *Mycobacterial* species directly from clinical specimens. J. Appl. Microbiol..

[15] Shi Y., Li B., Tao J., Cheng J., Liu H. (2021). The Complex Co-infections of Multiple Porcine Diarrhea Viruses in Local Area Based on the Luminex xTAG Multiplex Detection Method. Front. Vet. Sci..

[16] Anderson S., Wakeley P., Wibberley G., Webster K., Sawyer J. (2011). Development and evaluation of a Luminex multiplex serology assay to detect antibodies to bovine herpes virus 1, parainfluenza 3 virus, bovine viral diarrhoea virus, and bovine respiratory syncytial virus, with comparison to existing ELISA detection methods. J. Immunol. Methods.

[17] Cong F., Zhu Y., Wang J., Lian Y., Liu X., Xiao L., Huang R., Zhang Y., Chen M., Guo P. (2018). A multiplex xTAG assay for the simultaneous detection of five chicken immunosuppressive viruses. BMC Vet. Res..

[18] Laamiri N., Fällgren P., Zohari S., Ben Ali J., Ghram A., Leijon M., Hmila I. (2016). Accurate Detection of Avian Respiratory Viruses by Use of Multiplex PCR-Based Luminex Suspension Microarray Assay. J. Clin. Microbiol..

[19] van der Steen M. (2025). xMAP^®^ Technology Overview [EB/OL]. Diasorin & Luminex Support. https://support.diasorin.com/Blog/xmap-multiplexing/#top.

[20] Kumar A., Kaur J. (2014). Primer Based Approach for PCR Amplification of High GC Content Gene: *Mycobacterium* Gene as a Model. Mol. Biol. Int..

[21] Brennan P.C., Fritz T.E., Flynn R.J. (1969). Role of *Pasteurella pneumotropica* and *Mycoplasma pulmonis* in murine pneumonia. J. Bacteriol..

[22] Nadkarni M.A., Martin F.E., Jacques N.A., Hunter N. (2002). Determination of bacterial load by real-time PCR using a broad-range (universal) probe and primers set. Microbiology.

[23] Xu B., Chen X., Lu F., Sun Y., Sun H., Zhang J., Shen L., Pan Q., Liu C., Zhang X. (2021). Comparative Genomics of *Mycoplasma synoviae* and New Targets for Molecular Diagnostics. Front. Vet. Sci..

[24] Townsend K.M., Frost A.J., Lee C.W., Papadimitriou J.M., Dawkins H.J.S. (1998). Development of PCR assays for species- and type-specific identification of *Pasteurella multocida* isolates. J. Clin. Microbiol..

[25] Jeong O.M., Kang M.S., Blackall P.J., Jeon B.-W., Kim J.-H., Jeong J., Lee H.-J., Kim D.-W., Kwon Y.-K., Kim J.-H. (2020). Genotypic divergence of *Avibacterium paragallinarum* isolates with different growth requirements for nicotinamide adenine dinucleotide. Avian Pathol..

[26] Slana I., Kaevska M., Kralik P., Horvathova A., Pavlik I. (2010). Distribution of *Mycobacterium avium subsp. avium* and *M. a. hominissuis* in artificially infected pigs studied by culture and *IS901* and *IS1245* quantitative real time PCR. Vet. Microbiol..

[27] Babady N.E., Mead P., Stiles J., Brennan C., Li H., Shuptar S., Stratton C.W., Tang Y.-W., Kamboj M. (2012). Comparison of the Luminex^®^ xTAG^®^ RVP Fast Assay and the Idaho Technology FilmArray RP assay for detection of respiratory viruses in pediatric patients at a cancer hospital. J. Clin. Microbiol..

[28] Sambrook J., Russell D.W. (2001). Molecular Cloning: A Laboratory Manual.

[29] Xiao L., Wang Y., Kang R., Wu X., Lin H., Ye Y., Yu J., Ye J., Xie J., Cao Y. (2018). Development and application of a novel Bio-Plex suspension array system for high-throughput multiplexed nucleic acid detection of seven respiratory and reproductive pathogens in swine. J. Virol. Methods.

[30] Shrinet G., Chhabra R., Sharma A., Batra K., Talukdar S.J., Maan S. (2023). High throughput Luminex beads based multiplex assay for identification of six major bacterial pathogens of mastitis in dairy animals. Front. Cell. Infect. Microbiol..

[31] Battaglia A., Schweighardt A.J., Wallace M.M. (2011). Pathogen detection using a liquid array technology. J. Forensic Sci..

[32] Kuchipudi S.V., Yon M., Surendran Nair M., Byukusenge M., Barry R.M., Nissly R.H., Williams J., Pierre T., Mathews T., Walner-Pendleton E. (2021). A Highly Sensitive and Specific Probe-Based Real-Time PCR for the Detection of *Avibacterium paragallinarum* in Clinical Samples from Poultry. Front. Vet. Sci..

[33] Li K., Zhang Y., Luo T., Li C., Yu H., Wang W., Zhang H., Chen H., Xia C., Gao C. (2024). Development of a Triplex qPCR Assay Based on the TaqMan Probe for the Detection of *Haemophilus parasuis*, *Streptococcus suis Serotype 2* and *Pasteurella multocida*. Microorganisms.

[34] Sevilla I.A., Molina E., Elguezabal N., Li C., Yu H., Wang W., Zhang H., Chen H., Xia C., Gao C. (2015). Detection of *mycobacteria*, *Mycobacterium avium* subspecies, and *Mycobacterium tuberculosis* complex by a novel tetraplex real-time PCR assay. J. Clin. Microbiol..

[35] (2021). Laboratory Animals—Qualitative Analysis of Pathogen Nucleic Acids with Suspension Array Method.

[36] Periyannan Rajeswari P.K., Soderberg L.M., Yacoub A., Leijon M., Svahn H.A., Joensson H.N. (2017). Multiple pathogen biomarker detection using an encoded bead array in droplet PCR. J. Microbiol. Methods.

[37] Bao J., Wu Z., Ishfaq M., Miao Y., Li R., Clifton A., Ding L., Li J. (2020). Comparison of Experimental Infection of Normal and Immunosuppressed Chickens with *Mycoplasma gallisepticum*. J. Comp. Pathol..

[38] Lin L., Chen X.-L., Wu S.-H., Cai X., Jiang B., You W., Zheng M. (2025). Simultaneous Detection and Differentiation of Four *Eimeria* Species in Chickens (*E. tenella*, *E. maxima*, *E. necatrix*, and *E. acervulina*) Using a Multiplex TaqMan-MGB qPCR Assay. Animals.

[39] Al-baqir A., Hassanin O., Al-Rasheed M., Ahmed M.S., Mohamed M.H.A., El Sayed M.S., Megahed M., El-Demerdash A., Hashem Y., Eid A. (2023). *Mycoplasmosis* in Poultry: An Evaluation of Diagnostic Schemes and Molecular Analysis of Egyptian *Mycoplasma gallisepticum* Strains. Pathogens.

[40] Zhou L., Wang A., Song F., Wu J., Yu C., Wei S., Yang S., Zhang R., Jiang S., Zhu Y. (2025). Development of droplet digital PCR (ddPCR) for the detection and quantification of *Mycoplasma gallisepticum* in duck flocks. Microb. Pathog..

[41] Hnatow L.L., Keeler C.L., Tessmer L.L., Czymmek K., Dohms J.E. (1998). Characterization of MGC2, a *Mycoplasma gallisepticum* cytadhesin with homology to the *Mycoplasma pneumoniae* 30-kilodalton protein P30 and *Mycoplasma genitalium* P32. Infect. Immun..

[42] Zhao M., Wu W., Song W., Yang H., Liu H., Xie R., Huang X., Huang J., Hua L., Chen H. (2025). A quadruplex real-time fluorescent quantitative PCR detection method for identification and serotyping of *Pasteurella multocida*. Microb. Pathog..

[43] Pan Y., Yu Q., Wang Q., Li Q., Tian W., Xin L., Hu X., Xiao H., Liu Y., Zhu L.R.D. (2025). Establishment and epidemiological investigation of a dual fluorescent qPCR assay for *Pasteurella multocida* and Salmonella in yaks in the Tibetan Autonomous Prefecture of Garzê, China. Front. Cell. Infect. Microbiol..

[44] Anjaneya Singh S.D., Dhama K., Wani M., Gowthaman V., Chawak M. (2014). Molecular Characterization of *Avibacterium paragallinarum* Isolated from Poultry Flocks of India. Asian J. Anim. Vet. Adv..

[45] Šlosárek M. (1970). DNA base composition and adansonian analysis of *mycobacteria*. Folia Microbiol..

[46] Liu H., Pan S., Wang C., Yang W., Wei X., He Y., Xu T., Shi K., Si H. (2025). Review of respiratory syndromes in poultry: Pathogens, prevention, and control measures. Vet. Res..

[47] Wang M.J., Ma W.J., Pan Y., Chen J.X., Zhang H., Xia C.Y., Wang Y. (2024). Analysis of Major Vertically Transmissible Pathogens and Their Detection Standards in SPF Chickens. Lab. Anim. Comp. Med..

[48] Müştak İ., Kolukirik M. (2021). Development of Diagnostic Quantitative Real-Time PCR for *Mycoplasma gallisepticum* and *Mycoplasma synoviae*. Etlik Vet. Mikrobiyol. Derg..

[49] Guo M., Liu D., Chen X., Wu Y., Zhang X. (2022). Pathogenicity and innate response to *Avibacterium paragallinarum* in chickens. Poult. Sci..

