# Supplementary material for: A Multiplex Liquid Array Assay Based on Luminex xTAG Technology for the Detection of Five Respiratory Pathogens in SPF Chickens
Source: Vet Sci. 2026 Jul 29;13(8):753. doi: 10.3390/vetsci13080753 (PMC13517640; doi:10.3390/vetsci13080753)
Supplement: Supplementary file 1 [file vetsci-13-00753-s001.zip › vetsci-4449626-supplementary.pdf]

Supplementary Materials

The detailed primer screening and validation procedures.

For each target gene, multiple pairs of specific primers were designed using Primer 6.0 software, with amplicon sizes controlled between 100 and 200 bp. These primer pairs were initially validated by PCR amplification using genomic DNA from standard strains, followed by agarose gel electrophoresis to identify those with high amplification efficiency and single bands. The validated primer pairs were then gradually combined into the final five-plex system. Each time a new primer pair was added, its compatibility with the existing primer pairs was verified. When non-specific cross-amplification occurred, the problematic primer pairs were replaced and re-validated. In addition, the Multiple Primer Analyzer tool (Thermo Fisher Scientific) was used to analyze all primers and TAG sequences to ensure that dimer formation among primers and between primers and TAG sequences was controlled within acceptable limits. Through this stepwise screening and validation, the final specific primer pairs and TAG sequences for each target were determined and are listed in Table 2.

Table S1. Primer sequences used for adaptability verification.

Primer	Target gene	Accession number	Location	Sequence	Product size(bp)	MagPlex-TAG™ Microsphere Part Numbers	Reference
MG-F2	*mgc2*	LS991952.1	220490~220512	CAAATACATAATCTTACATTCACT/iSp18/TACGAACATTCACCCACACTTGT	110	13	[10]
MG-R2			220581~220599	5`Biotin - CCAGCACCTGCACCCACTA			
PM-F2	*kmt1*	CP008918.1	202958~202980	TACTTCTTTACTACAATTTACAAC/iSp18/TTGACAGCTTTGTGATCTGGATTG	66	15	[10]
PM-R2			203002~203023	5`Biotin - GTCACTCTACTGGCGCGTTAAA			
Apg-F2	*recN*	DQ899748.1	584~603	AATCTCTACAATTTCTCTCTAATA/iSp18/C TCACAAACCTTTCGCAATCG	85	61	[10]
Apg-R2			649~668	5`Biotin - TGGATTGTGCGGTAGAGCAA			
MG-F3	*16S rRNA*	LS991952.1	312721~312740[a]	CAAATACATAATCTTACATTCACT/iSp18/ATCTAAAACCGTGTTGCTAA	113	13	[35]
MG-R3			312815~312833	5`Biotin - AGGAGGTAATCCACCCCCA			
MS-F2	*16S rRNA*	CP011096.1	701795~701816	ATACTTTACAAACAAATAACACAC/iSp18/CCTTTTAGACTGGAATAACGGT	121	19	[35]
MS-R2			701696~701712	5`Biotin - GCTAATGTTCCGCACCC			
Mav-F2	*IS1245*	L33879.1	67~84	CTAAACATACAAATACACATTTCA/iSp18/GTGAGTCTCTGTGGTGAA	161	62	This study
Mav-R2			210~217	5`Biotin - CGATCAAGGCTTGGTAGA			

Note: 'This study' indicates the primer pair designed in the present work.

[10] Wang H, Xue L, Wang L, et al. The quadruplex fluorescent quantitative PCR method for the simultaneous detection of respiratory diseases in quail: Pasteurella multocida, Avibacterium paragallinarum, Mycoplasma gallisepticum, and Mycoplasma synoviae. Front Microbiol. 2025;16:1605356.

[35] Guangdong Provincial Administration for Market Regulation. Laboratory animals — Qualitative

analysis of pathogen nucleic acids with suspension array method. DB44/T 2338-2021. 2021.

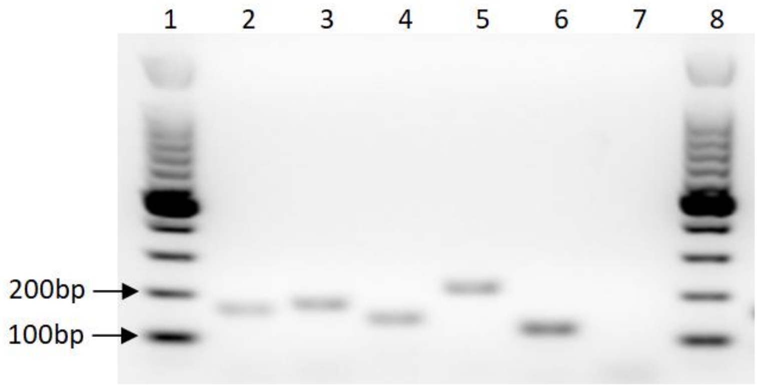

Figure S1. Agarose gel electrophoresis verification of the pentaplex PCR products. 1 and 8: 100 bp DNA Marker; 2-6: MG, MS, Pm, Apg and Mav; 7: Negative control.

Table S2. Specificity test Results (partial, MFI values).

Stains	Source	MagPlex-TAG™ Microsphere Part Numbers and xTAG assay MFI values				
		**No. 13**	**No. 15**	**No. 19**	**No. 61**	**No. 62**
Mycoplasma pulmonis	ATCC 19612	74.5	90.0	64.0	70.0	68.5
Mycoplasma pneumoniae	ATCC 15521	66.0	69.0	69.0	58.5	74.0
Mycoplasma orale	ATCC 23714	54.0	72.0	64.0	72.5	57.0
Mycoplasma neurolyticum	ATCC 15049	72.5	82.0	65.5	72.5	69.0
Mycoplasma arthritidis	ATCC 19611	61.0	98.0	64.0	66.5	59.0
Rodentibacter pneumotropicus	ATCC 35149	62.0	70.0	66.0	67.0	63.5
Rodentibacter caecimuris	ATCC 12555	79.0	77.0	72.0	59.0	59.0
Pasteurella aerogenes	ATCC 27883	69.0	77.0	70.5	65.0	70.0
Actinobacillus muris	ATCC 49577	66.5	78.0	77.5	72.0	66.5
Bordetella bronchiseptica	ATCC 19395	63.0	64.5	61.0	69.5	68.0
Streptococcus pneumoniae	CMCC 33210	66.0	75.5	62.0	76.5	75.0
Streptococcus pyogenes	CMCC 32210	65.0	69.0	75.0	67.0	76.0
Avibacterium gallinarum	ATCC 13361	61.5	64.5	71.0	66.0	74.0
Avibacterium avium	ATCC 29546	65.0	85.0	81.0	65.5	63.5
Haemophilus haemolyticus	ATCC 33390	62.0	61.0	63.0	57.5	64.5
Haemophilus influenzae	ATCC 33391	73.0	74.0	69.0	71.5	79.0
Mycobacterium tuberculosis	CMCC 93009	58.0	85.0	75.0	78.0	70.5
Mycobacterium bovis	CMCC 95055	53.0	87.0	65.0	79.0	58.0
MG	CVCC 352	2085.5	95.0	95.0	74.0	79.0
MS	ATCC 25204	63.0	96.0	2003.0	74.0	79.0
Pm	ATCC 43137	65.0	2721.0	75.0	81.0	136.0
Apg	ATCC 29545	80.0	64.0	77.0	2452.5	76.5
Mav	CMCC 95001	78.5	62.0	93.0	75.5	1985.0
Mixed five targetgenomic DNA derived from the strains	/	471.5	897.0	417.0	446.0	361.5
NTC	/	11.0	31.5	20.0	42.5	13.5

Table S3. Sample Detection Results (partial, MFI values).

No. Sample	No. Microsphere and MFI values				
	**No. 13**	**No. 15**	**No. 19**	**No. 61**	**No. 62**
1	22.0	19.5	24.5	34.0	29.0
2	40.0	55.5	23.0	28.0	17.0
3	26.0	41.0	32.0	17.0	22.0
4	25.0	44.0	15.5	23.0	25.0
5	31.0	15.0	23.0	27.5	27.0
6	9.5	68.0	37.0	15.0	41.0
7	26.0	25.0	16.0	15.0	23.0
8	13.5	32.0	46.0	31.0	29.0
9	31.0	42.0	37.0	9.0	36.5
10	25.0	22.5	48.0	18.0	68.0
11	18.0	18.0	25.0	24.0	16.0
12	19.0	18.0	17.0	19.5	19.0
13	5.0	26.0	14.0	20.0	22.0
14	11.5	15.5	31.0	14.0	18.0
15	15.0	10.0	23.0	20.0	19.0
16	25.0	25.0	23.5	26.5	28.5
17	22.0	19.0	6.5	20.0	15.0
18	10.5	25.0	25.5	29.0	21.0
19	24.5	29.0	26.5	15.5	18.5
20	9.5	22.0	18.5	23.5	21.5
PC	398.0	864.0	417.0	446.0	361.5
NC	11.0	31.5	20.0	42.5	13.5

Table S4. Adaptability Test Results of Previously Reported qPCR Primers on the Luminex Platform.

Combination	DNA dilution	No. Microsphere and MFI values		
		No. 13	No. 15	No. 61
Wang et al. reported a multiplex real-time PCR method combining *MG, Pm*, and *Apg*.	10^{-1}	4933.50	3944.00	3652.00
	10^{-2}	5261.75	4054.00	3462.50
	10^{-3}	5262.25	4312.75	2559.50
	10^{-4}	4026.75	4419.75	1326.00
	10^{-5}	3392.00	4822.00	559.75
	NC	2613.75	5278.00	636.00

Table S5. Combination Test Results of Previously Reported qPCR Primers with the Primers of the Present Study.

Combination	DNA dilution	No. Microsphere and MFI values				
		No. 13	No. 15	No. 19	No. 61	No. 62
The *MG, Pm,* and *Apg* primers (Wang et al.) were combined with the *MS* and *Mav* primers (this study).	10^{-2}	5095.0	3944.0	468.0	3313.5	2019.0
	10^{-3}	2703.0	3184.0	225.0	1081.0	1045.0
	10^{-4}	1875.0	4218.0	97.0	421.0	1199.0
	10^{-5}	613.5	3014.5	100.5	127.0	1253.0
	10^{-6}	91.0	1544.5	123.0	75.0	1531.0
	NC	94.0	2112.0	155.0	155.5	1784.0

Table S6. Replacement Test Results Using Previously Reported Liquid Array Primers.

Combination	DNA dilution	No. Microsphere and MFI values				
		No. 13	No. 15	No. 19	No. 61	No. 62
The *MG* and *MS* primers (Guangdong Provincial Local Standard) were combined with the *Pm, Apg,* and *Mav* primers (this study).	10^{-1}	3414.0	1925.5	2275.0	1726.0	3515.0
	10^{-2}	3782.5	2246.5	1917.0	1557.0	3371.5
	10^{-3}	2282.0	2384.0	1177.5	1206.0	3527.0
	10^{-4}	1540.0	2231.0	253.0	814.0	3024.0
	10^{-5}	587.0	2819.5	533.0	1169.0	4208.0
	10^{-6}	371.5	2255.0	95.0	1028.0	3156.0
	NC	1283.0	2524.0	1538.0	1287.0	3862.0

Table S7. Replacement Test Results Using Alternative Mav Primers.

**Template Concentration of pUC57-IS1245 （copies/μL）**	**Mav-F/R（this study）**	**Mav-F2/R2**
10^5	7128.0	8513.0
10^4	5994.5	8895.5
10^3	5271.5	7591.5
10^2	1985.0	5506.0
10^1	388.5	4707.5
10^0	211.5	4178.5
10^{-1}	77.5	2409.5
Genomic DNA of *Mav* (CMCC 95001)	2126.0	1769.5
NTC	83.0	397.0

Table S8. Summary of analytical performance metrics of the developed multiplex luminex array assay.

**Performance metric**	***MG***	***MS***	***Pm***	***Apg***	***Mav***
LOD (copies/μL)	100	100	10	100	100
Linear range (copies/μL)	10^2–10^6	10^2–10^6	10^2–10^6	10^2–10^6	10^2–10^6
Intra-assay CV (%) range	0.8–6.3	2.1–8.6	5.5–8.5	1.7–10.5	2.8–12.2
Inter-assay CV (%) range	2.3–10.4	4.8–13.0	11.3–14.4	3.1–13.0	3.0–8.3
Specificity (non-targets tested)	18/18	18/18	18/18	18/18	18/18
concordance vs. qPCR	79.8%	100%	100%	100%	100%
